# Early Supera stent fracture after treatment of popliteal artery occlusion caused by artificial knee joint: a case report

**DOI:** 10.1093/ehjcr/ytaf114

**Published:** 2025-04-04

**Authors:** Yuki Shima, Narumi Taninobu, Kazunori Mushiake, Hiroyuki Tanaka, Kazushige Kadota

**Affiliations:** Department of Cardiovascular Medicine, Kurashiki Central Hospital, 1-1-1 Miwa, Kurashiki 710-8602, Japan; Department of Cardiovascular Medicine, Kurashiki Central Hospital, 1-1-1 Miwa, Kurashiki 710-8602, Japan; Department of Cardiovascular Medicine, Kurashiki Central Hospital, 1-1-1 Miwa, Kurashiki 710-8602, Japan; Department of Cardiovascular Medicine, Kurashiki Central Hospital, 1-1-1 Miwa, Kurashiki 710-8602, Japan; Department of Cardiovascular Medicine, Kurashiki Central Hospital, 1-1-1 Miwa, Kurashiki 710-8602, Japan

**Keywords:** Peripheral artery disease, Endovascular therapy, Stent fracture, Case report

## Abstract

**Background:**

The Supera stent, a self-expanding interwoven nitinol stent, has greater radial force than conventional stents, resulting in high patency and reduced stent fracture. There have been few reports of early stent fracture, and it is considered an effective device in the popliteal artery.

**Case summary:**

We present a case of popliteal artery occlusion due to Supera stent fracture. An 86-year-old woman presented to our department with intermittent claudication after 6 months of Supera stent implantation. Ultrasonography demonstrated popliteal artery occlusion secondary to Supera stent fracture. Fluoroscopy revealed that the stent had fractured at the site closest to the artificial knee joint and might have been in contact with a vessel. In addition, the popliteal artery was slightly deviated medially in the P2 segment, suggesting popliteal artery entrapment syndrome. Because additional stenting was considered a risk for further stent failure, it was decided to complete endovascular treatment with balloon angioplasty alone and then consider bypass treatment.

**Discussion:**

Although the Supera stent is a useful stent, it would be prudent to avoid popliteal artery stenting in the presence of abnormal vascular running or artificial joint is observed.

**Learning points**

- To identify risk factors associated with deploying Supera stent fracture in popliteal artery.
- To recognize if the vascular run of the popliteal artery is normal or not.

## Introduction

Owing to its repetitive flexion, the popliteal artery is a challenging anatomic site for the endovascular treatment of peripheral artery disease. The popliteal artery is considered a non-stenting zone because the mobility of the knee joint introduces additional dynamic forces into the vessel, resulting in accelerated restenosis and higher rates of stent fracture and occlusion. Therefore, the use of self-expanding nitinol stents in the popliteal artery has been avoided.^1^ The Supera stent (Abbott Vascular, Santa Clara, CA, USA) is a self-expanding interwoven nitinol stent that offers greater resistance to fracture and kinking, radial force, and flexibility than other self-expanding nitinol stents.^2^ As the Supera stent has been shown to have excellent primary patency and a low stent fracture rate at mid- and long-term follow-ups, popliteal artery stenting is becoming more common.^3,4^ As early Supera stent fracture is rare and has been scarcely reported, we present a case of popliteal artery occlusion due to Supera stent fracture at six months postoperatively.

## Summary figure

**Figure:**
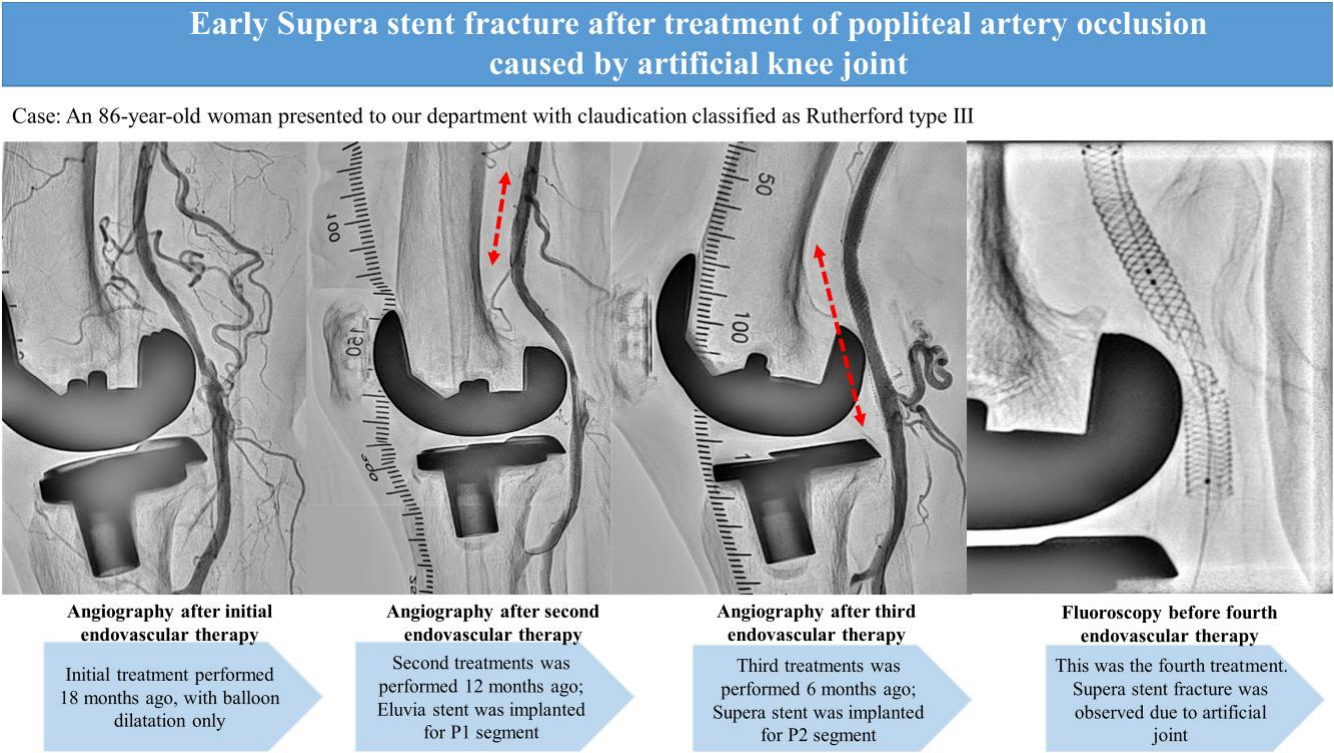


## Case presentation

**Figure 1:**
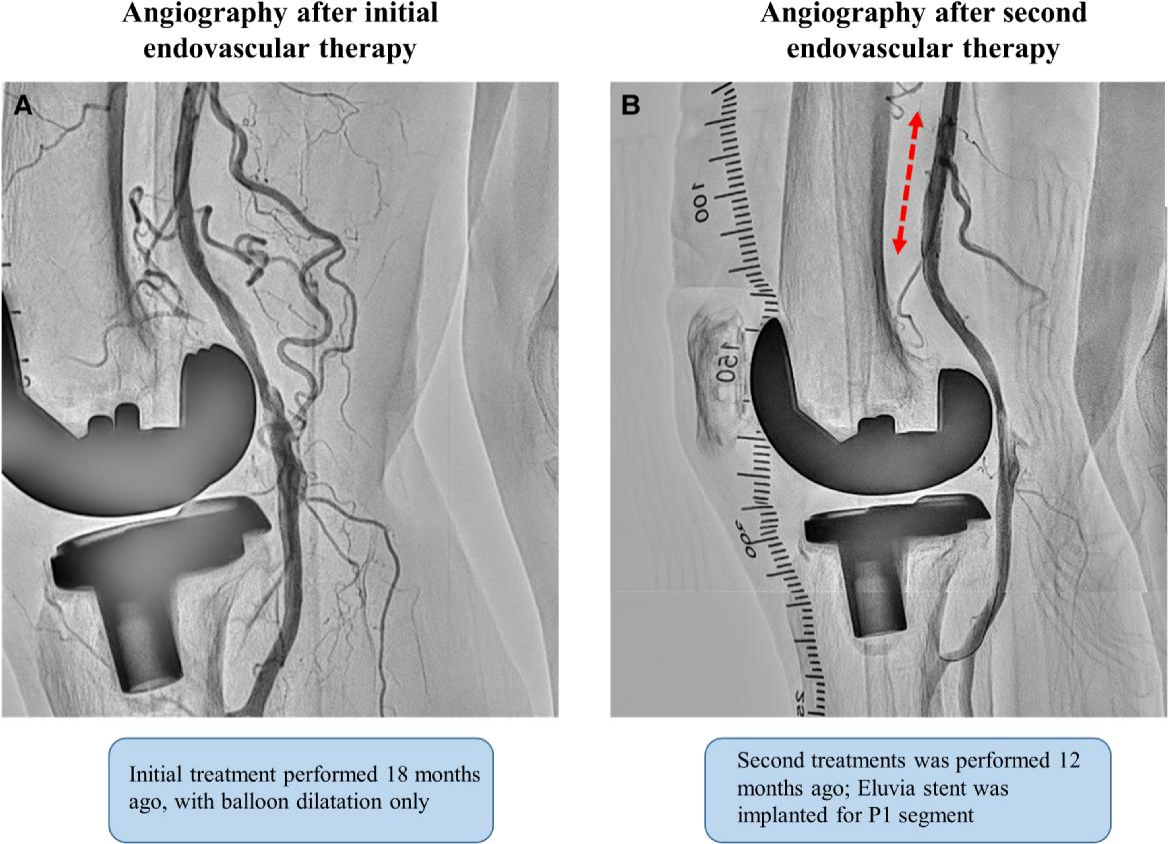
First and second endovascular treatment (EVT) angiograms. (*A*) The first EVT was completed with balloon angioplasty only. (*B*) The second EVT was Eluvia stenting in the P1 segment and balloon angioplasty alone in the P2 segment.

**Figure 2:**
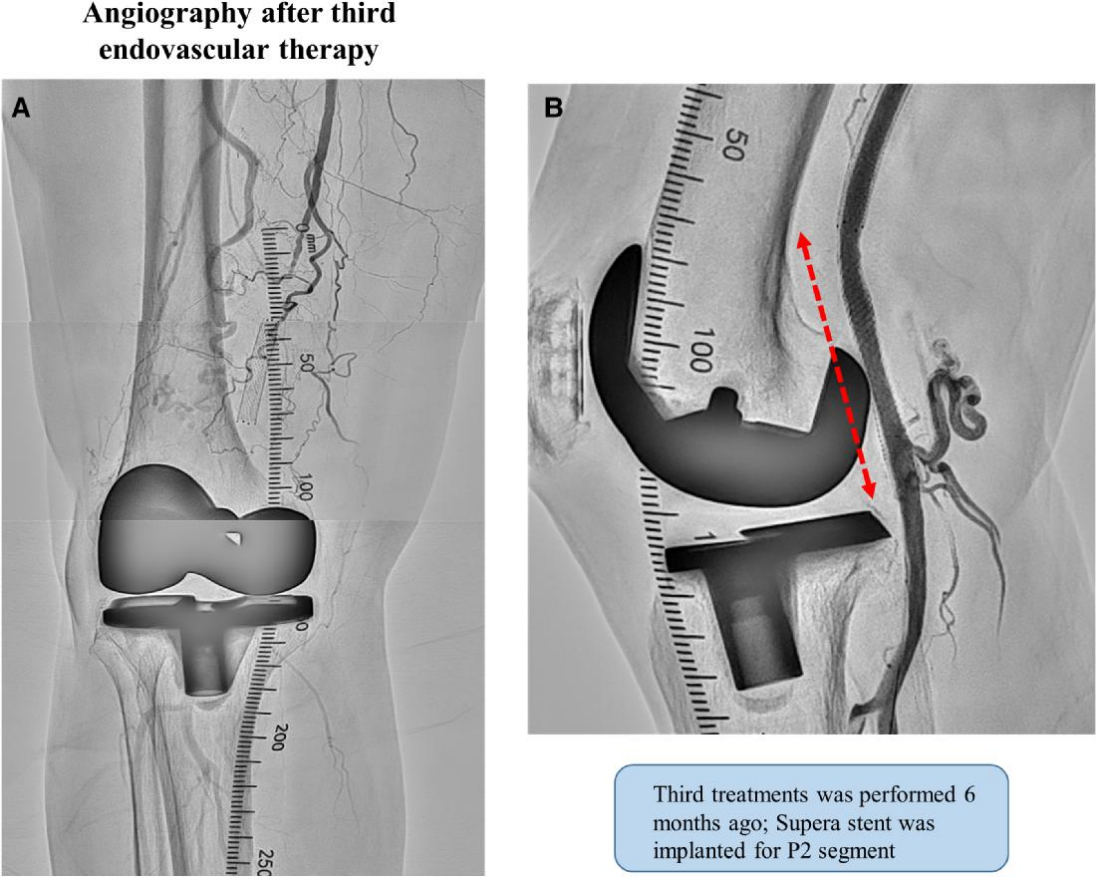
Third endovascular treatment angiograms. (*A*) Occluded lesions over superficial femoral artery to popliteal artery. (*B*) Supera stent was deployed, and blood flow was good.

An 86-year-old woman presented to our department with intermittent claudication classified as Rutherford type Ⅲ. She was diagnosed with peripheral artery disease because the right popliteal artery pulse was not palpable. She had hypertension, hyperlipidaemia, and a history of total knee arthroplasty years before the initial medical examination. Lower extremity ultrasonography revealed an occlusion of the P1 and P2 segments of the popliteal artery. The patient was an older adult and requested to be treated by endovascular therapy (EVT), which was performed using balloon dilation alone. (*Figure 1A*). However, at 6 months postoperatively, the patient redeveloped intermittent claudication symptoms. The distal part from the superficial femoral artery (SFA) to the P2 segment of the popliteal artery was re-occluded, and the patient requested another EVT to improve symptoms. During the second treatment, balloon dilation was performed, however, a large amount of thrombosis was noted at the distal part of SFA, and blood flow could not be obtained. Therefore, Eluvia 6.0 × 40 mm (Boston Scientific, Marlborough, MA, USA) stenting was performed in the distal SFA, and P1–P2 segments were dilated using Ranger 5.0 × 60 mm (Boston Scientific, Marlborough, MA, USA), as a drug-coated balloon (*Figure 1B*). The P2 segment was slightly poorly dilated, however, as the lesion was in a non-stenting zone, treatment was considered complete. However, at 6 months after the second endovascular treatment, the popliteal artery re-occluded and the patient redeveloped intermittent claudication symptoms. Therefore, a third endovascular treatment was performed. At this time, the lesion was also thrombotic, and the P2 segment was poorly dilated. Because of the possibility of another re-occlusion if the lesion remained poorly dilated, Supera stenting was performed with the patient’s consent to achieve long-term patency (*Figure 2*). Postprocedural intravascular ultrasound showed no evidence of stent compression.

**Figure 3:**
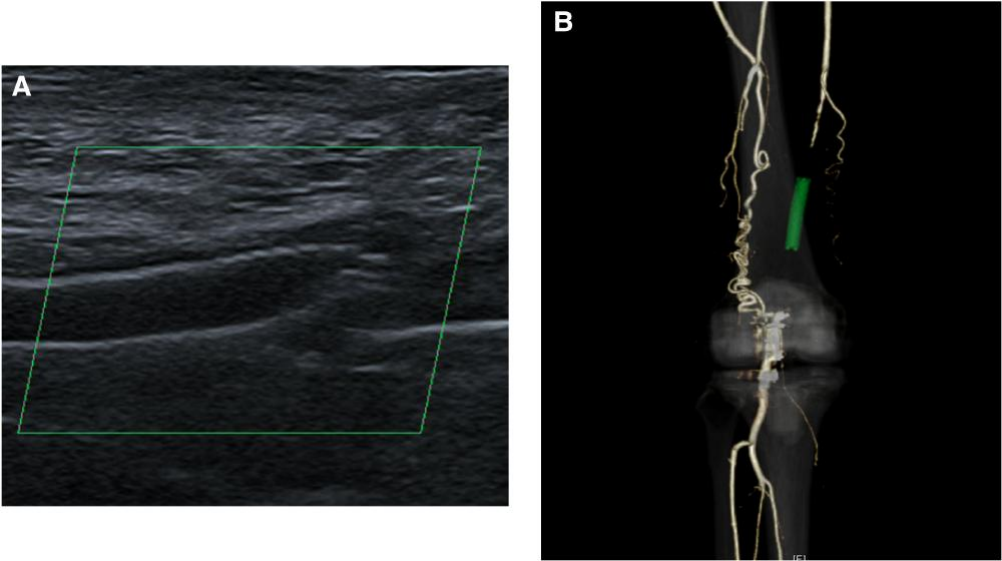
Imaging studies six months after the third endovascular treatment. (*A*) Ultrasonography demonstrated popliteal artery occlusion secondary to Supera stent fracture. (*B*) Contrast-enhanced computed tomography scan showed occlusion from the superficial femoral artery to the popliteal artery despite the development of collateral blood channels.

**Figure 4:**
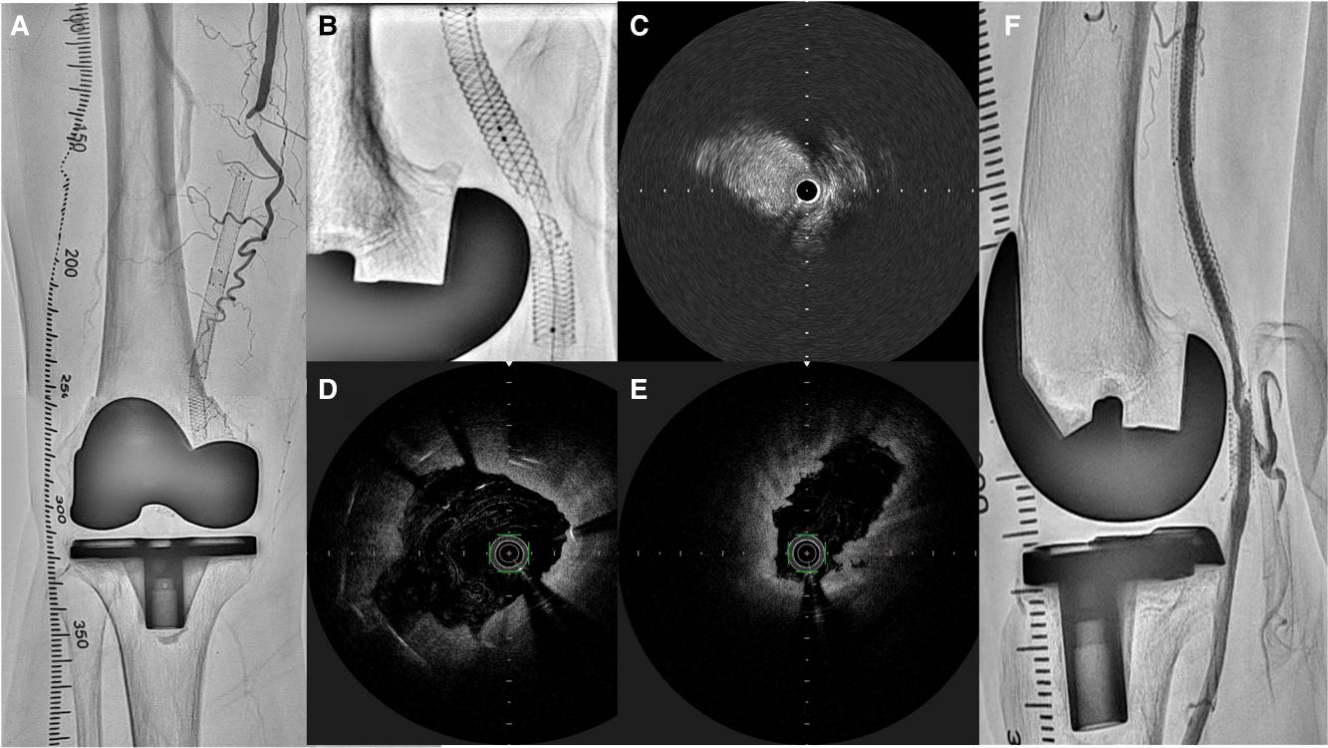
Imaging studies of the fourth endovascular treatment. (*A*) Angiography showed occlusion from the superficial artery to the popliteal artery. (*B*) Fluoroscopy showed Supera stent fracture at the site closest to the artificial knee joint. (*C*) Optical frequency domain imaging demonstrated Supera stent fracture with partial protrusion into the lumen and (*D*) areas not covered by the stent. (*E*) Intravascular ultrasound showed a lesion with low stent coverage and vessel dilation. (*F*) Final angiography confirmed blood flow, but vessel dilation was inadequate.

At six months following the third endovascular treatment, the patient returned to our department with intermittent claudication and loss of distal and popliteal pulses in the right lower limb. The patient’s intermittent claudication symptoms became progressively worse with each recurrence, and this time the symptoms appeared after ∼100 m of walking. Her ankle-brachial index was 0.66. Ultrasonography demonstrated popliteal artery occlusion secondary to Supera stent fracture (*Figure 3A*). The lesion repeatedly re-occluded, so a contrast-enhanced computed tomography scan was performed to evaluate the possibility of popliteal artery entrapment syndrome, which showed occlusion from the SFA to the popliteal artery despite the development of collateral blood channels (*Figure 3B*). Furthermore, angiography showed occlusion from the superficial artery to the popliteal artery (*Figure 4A*).

The fourth endovascular treatment was performed for popliteal artery occlusion. A 45-cm 6 F Destination guide catheter (Terumo, Tokyo, Japan) was inserted into the right common femoral artery via an antegrade approach. Fluoroscopy showed stent fracture at the site closest to the artificial knee joint, which appeared to be in contact with the vessel (*Figure 4B*). A 0.014-inch Gladius guidewire (Asahi Intecc, Seto, Japan) was supported by a Prominent Advance Neo 90-cm microcatheter (Tokai Medical Products, Kasugai, Japan) and successfully crossed the occluded lesion. Optical frequency domain imaging demonstrated Supera stent fracture with partial protrusion into the lumen and areas not covered by the stent, but no evidence of stent compression by the artificial knee joint (*Figure 4C* and *D*). Intravascular ultrasound showed a lesion with low stent coverage and vessel dilatation (*Figure 4E*). The lesion was dilated with a 3.0 × 40 mm JADE PTA balloon catheter (OrbusNeich Medical Tokyo, Japan) and a 5.0 × 40 mm Aperta PTA balloon catheter (Nipro, Osaka, Japan). Final angiography confirmed blood flow was re-established, but vessel dilation remained inadequate (*Figure 4F*). First and final angiography videos showed in Supplementary material. However, re-stenting could result in another stent fracture, therefore bypass surgery was considered. She was started on aspirin 100 mg/day and clopidogrel 75 mg/day as dual anti-platelet therapy. Other medications were pitavastatin 2 mg/day o.d. and azilsartan 10 mg/day o.d. These medications were taken regularly from the first to the last treatment. Because of repeated re-occlusion, the anti-platelet therapy plan was to continue dual anti-platelet therapy for at least one year. The patient’s symptoms did not improve and she requested surgical treatment, so bypass surgery was performed three months after the last EVT. The lesion showed good patency for the six months after bypass surgery.

## Discussion

To our knowledge, this is the first case of early Supera stent fracture and there are two possible causes exist. First, the Supera stent may have fractured due to physical irritation caused by contact with the artificial knee joint. Optical frequency domain imaging and intravascular ultrasound showed no evidence of stent compression, but the stent fracture occurred in close proximity to the artificial knee joint; it is highly likely that the stent was physically affected by joint flexion or other stresses. Second, Supera stent fracture may have occurred due to popliteal artery entrapment syndrome. This is because the popliteal artery is repeatedly compressed by abnormal attachments of the gastrocnemius muscle or by abnormal muscles and fibre bundles in the popliteal fossa, resulting in impaired blood flow. It is more common in men under the age of 30 and may be especially common in athletes with high muscle mass.^5^ In this case, there were no abnormal attachments or abnormal muscle bundles in the popliteal fossa on the computed tomography scan, although they could not be fully evaluated due to artefacts from the artificial knee joint. The popliteal artery appeared to be slightly deviated medially in the P2 segment, and suggesting abnormal vascular running. The patient has atherosclerotic factors such as hypertension and hyperlipidaemia, but very mild atherosclerosis in the left lower extremity, aorta, and coronary arteries. Thus, the combination of abnormal vascular running and the artificial knee joint may have caused early Supera stent fracture. There are case reports of Supera stenting in an occluded popliteal artery with stent fracture.^6,7^ In this case, the risk of stent fracture was considered high even with additional Supera stenting, and no additional stenting was performed.

Even with the advent of the Supera stent, avoiding stenting in non-stenting zones remains common practice. However, there are situations in which stenting is unavoidable, such as thrombotic lesions and severe dissection. In addition, the strong radial force of the Supera stent is particularly useful in calcified lesions. Previous studies have reported that the Supera stent demonstrated good patency at two years without stent fracture^8^ and good patency at one year without stent fracture in popliteal artery lesions.^9^ In the BURDOCK study, the one-year and two-year patency rates of the Supera stent in calcified femoropopliteal lesions were 88.2% and 80.8%, respectively,^10^ suggesting that the Supera stent is a suitable device for femoropopliteal lesions.

## Conclusion

The Supera stent is an excellent stent, however, avoiding popliteal artery stenting if a suspicion of abnormal vascular running or an artificial joint is observed may be prudent.

## Supplementary Material

ytaf114_Supplementary_Data

## Data Availability

The data underlying this article cannot be shared publicly due to the privacy of individuals that participated in the study.

## Lead author biography

The lead author graduated from Nihon University in 2015 and currently work as a cardiologist at Kurashiki Central Hospital, Japan.

## Notes

**Consent:** The authors confirm that written consent for submission and publication of this case report, including the images and associated text, has been obtained from the patient in accordance with COPE guidance.

**Funding:** We have nothing to declare.
